# Aluminum doping tunes band gap energy level as well as oxidative stress-mediated cytotoxicity of ZnO nanoparticles in MCF-7 cells

**DOI:** 10.1038/srep13876

**Published:** 2015-09-08

**Authors:** Mohd Javed Akhtar, Hisham A. Alhadlaq, Aws Alshamsan, M.A. Majeed Khan, Maqusood Ahamed

**Affiliations:** 1 King Abdullah Institute for Nanotechnology, King Saud University, Riyadh 11451, Saudi Arabia; 2 Department of Physics and Astronomy, College of Science, King Saud University, Riyadh 11451, Saudi Arabia; 3 Nanomedicine Research Unit, Department of Pharmaceutics, College of Pharmacy, King Saud University, Riyadh 11451, Saudi Arabia

## Abstract

We investigated whether Aluminum (Al) doping tunes band gap energy level as well as selective cytotoxicity of ZnO nanoparticles in human breast cancer cells (MCF-7). Pure and Al-doped ZnO nanoparticles were prepared by a simple sol-gel method. Characterization study confirmed the formation of single phase of Al_x_Zn_1-x_O nanocrystals with the size range of 33–55 nm. Al-doping increased the band gap energy of ZnO nanoparticles (from 3.51 eV for pure to 3.87 eV for Al-doped ZnO). Al-doping also enhanced the cytotoxicity and oxidative stress response of ZnO nanoparticles in MCF-7 cells. The IC50 for undoped ZnO nanoparticles was 44 μg/ml while for the Al-doped ZnO counterparts was 31 μg/ml. Up-regulation of apoptotic genes (e.g. p53, bax/bcl2 ratio, caspase-3 & caspase-9) along with loss of mitochondrial membrane potential suggested that Al-doped ZnO nanoparticles induced apoptosis in MCF-7 cells through mitochondrial pathway. Importantly, Al-doping did not change the benign nature of ZnO nanoparticles towards normal cells suggesting that Al-doping improves the selective cytotoxicity of ZnO nanoparticles toward MCF-7 cells without affecting the normal cells. Our results indicated a novel approach through which the inherent selective cytotoxicity of ZnO nanoparticles against cancer cells can be further improved.

Nanotechnology represents a unique platform that promises to provide improved technologies for biological applications. This new technology allows the controlled manipulation of materials/devices at nanoscale level (1–100 nm). Nanoscale materials are on the same size scale as biological molecules, and so are better able to penetrate cells and interact with biomolecules where larger molecules have limited accessibility^1^. The reduction of materials to the nano-scale can frequently alter their optical, electrical, magnetic, structural and chemical properties enabling them to interact in a unique way with biological systems^2^.

ZnO nanoparticles have multiple properties including favorable band gap, electrostatic charge, surface chemistry and potentiation of redox-cycling cascades^3^. These characteristics of ZnO nanoparticles are being exploited in biomedical field such as cell imaging, bio-sensing and drug delivery. Recently, ZnO nanoparticles have received much attention for their potential application in cancer therapy. One of the primary advantages for considering ZnO nanoparticles in cancer therapy is their inherent preferential cytotoxicity against cancer cells. Our previous studies have shown that ZnO nanoparticles selectively kill human lung and liver cancer cells while posing no toxicity to normal cells^4^. Ostrovsky *et al.*^5^ have shown that ZnO nanoparticles exerted cytotoxic effect on several human glioma cells and no cytotoxic effect was observed on normal human astrocytes. ZnO nanoparticles exhibited a preferential ability to kill human myeloblastic leukemia cells (HL60) as compared with normal peripheral blood mononuclear cells^6^. Hanley *et al.*^7^ also observed that ZnO nanoparticles exhibit a strong preferential ability to kill cancerous T cells compared with normal cells. These data suggest that ZnO nanoparticles have potential to develop as an anticancer candidate. However, for practical therapeutic applications, new strategies are required to further improve the cancer killing ability of ZnO nanoprticles without affecting normal cells. This study focuses on improving the cancer cells killing ability of ZnO nanoparticles by metal ions doping.

ZnO is a conventional wide band-gap semiconductor and is currently under investigation owing to its tremendous applications in response to its tunable properties. Wide band-gap semiconductor properties of ZnO nanoparticles are also useful in induction of intracellular reactive oxygen species (ROS) generation^8^. Conduction electrons (e^−^) and valence holes (h^+^) in semiconductors have been traditionally used for photocatalytic oxidation of organic and inorganic pollutants, and as sensitizers for the photodestruction of cancer cells through oxidative damage of biomolecules^9^. However, in those experiments, adequate electrons and holes were typically produced via UV irradiation and excitation. In case of ZnO nanoparticles large numbers of holes and/or electrons could be available even without the presence of UV light. Sakthivel *et al.*^10^ has demonstrated that ZnO can comparatively absorb more light than TiO_2_ in the region where the light absorption occurs due to band gap excitation. The study further suggests that, the optical absorption of ZnO can be enhanced by creating more defects (e.g. metal ions doping) on its surface. Our previous study demonstrated that production of electrons and/or holes on ZnO nanoparticles surface can be increased by Aluminum (Al) ions doping^11^. Various activated oxygen species can be produced by the reactions of holes and electrons in a metal oxide semiconductor^12^,^13^. Therefore, a fundamental understanding on the optical properties of ZnO nanoparticles becomes crucial for their application in cancer therapy.

The underlying mechanisms of cytotoxicity of ZnO nanoparticles are not fully explored. However, intracellular generation of ROS due to ZnO nanoparticles exposure is believed to play major role. When nanoparticles interact with cells, cellular defense mechanisms are activated to minimize the damage. Nevertheless, if ROS production exceeds the antioxidants defense capacity of the cell, it results in oxidative damage of biomolecules, which can lead to cell death^14^. Our recent studies^15^,^16^ as well as others^7^,^12^ have reported that ROS play a critical role in ZnO nanoparticles induced apoptosis. It is also suggested that metal ions doping on metal oxides have high catalytic activity in generating ROS, and in oxidizing cellular macromolecules^17^.

Based on earlier literature and our previous research^4^,^11^,^12^, this study was designed to evaluate whether Al ions doping tunes band gap energy level as well as cytotoxicity via ROS generation of ZnO nanoparticles in human breast cancer cells (MCF-7). We further explore the underlying mechanisms of apoptosis induced by Al-doped ZnO nanoparticles in MCF-7 cells. We have chosen MCF-7 cells because the breast cancer is a severe and life threatening cancer and the incidence of such type of cancer is increasing at an alarming rate worldwide^18^. This cell line (MCF-7) has also been widely used in toxicological and pharmacological studies^19^,^20^,^21^.

## Results

### XRD characterization of nanoparticles

**Figure 1:**
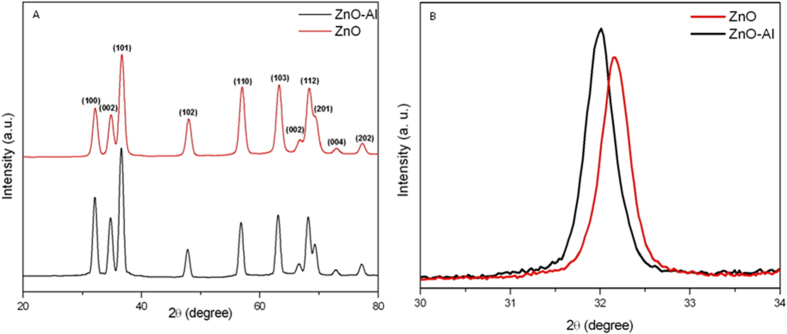
(**A**) XRD pattern of pure and Al-doped ZnO nanoparticles. (**B**) XRD pattern (zoom) corresponding to peak (101) of pure and Al-doped ZnO nanoparticles.

Figure 1A represents the XRD pattern of the pure and Al-doped ZnO nanoparticles. XRD pattern shows that Al-doping did not change the hexagonal wurtzite structure (JCPDS89-0510) of ZnO nanoparticles and suggested the formation of single phase of Al_x_Zn_1-x_O. Broadening of diffraction peaks due to Al-doping was observed that indicated the decrease in nanoparticles size. As we can see in Fig. 1B, ZnO nanoparticles peak corresponding to (101) plane shifted slightly to lower angle due Al-doping. Shifting of peak could be due to incorporation of dopant ions into the lattice of the host material. Similar finding were reported by other investigators^11^,^22^. The crystallite size (D) of pure and Al-doped ZnO nanoparticles was calculated using Scherrer’s formula (equation 1)^11^.


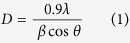


Where β is FWHM of a peak and λ is the wavelength of incident X-rays. Crystallite size of both samples was calculated corresponding to most intense peak (101). Crystallite size of pure and Al-doped ZnO nanoparticles were 41 and 39 nm, respectively (Table 1).

### TEM characterization of nanoparticles

**Figure 2:**
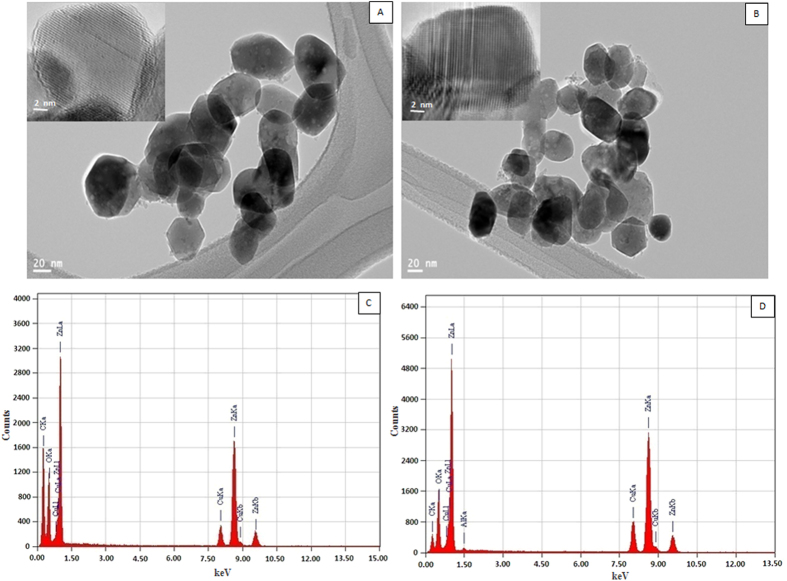
TEM images of pure (**A**) and Al-doped (**B**) ZnO nanoparticles. Inset shows HR-TEM of same samples. EDS analysis of pure (**C**) and Al-doped (**D**) ZnO nanoparticles.

Morphology of pure and Al-doped ZnO nanoparticles was investigated by FETEM. Figure 2A,B represent the micrographs of pure and Al-doped ZnO nanoparticles, respectively. These images demonstrated that grown nanoparticles were almost spherical shaped with the particle size range of 33–55 nm. High resolution TEM images represented at insets showing that particles nature were crystalline that supports XRD data. TEM average diameter was calculated from measuring over 100 particles in random fields of view. The average particle size of pure and Al-doped ZnO nanoparticles were approximately 43 and 40 nm, respectively. The average particle size obtained from TEM matches well with the size estimated from the XRD. Both TEM and XRD studies showed a slight decrease in ZnO nanoparticles size with Al-doping. This is a common trend when a dopant is incorporated into ZnO particles and supported by other studies^11^,^23^. The size reduction of Al-doped ZnO samples may be owing to smaller ionic radius of Al compared to ionic radius of Zn.

The self-generated elemental composition details are presented in EDS pattern given in Fig. 2C,D. Additional peaks assigned to C and Cu are due to the carbon coated copper grid. It is clear from these images that Zn and O were the main elemental species in pure ZnO samples while additional Al peak was observed in Al-doped samples.

### Optical properties of nanoparticles

**Figure 3:**
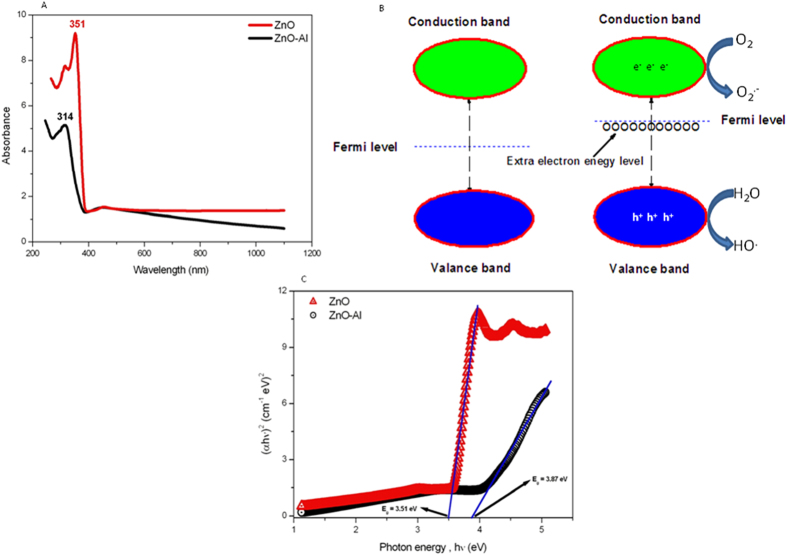
(**A**) UV-visible absorption spectra of pure and Al-doped ZnO nanoparticles. (**B**) Shifting of Fermi level due to transfer of electrons from valence band to conduction band. (**C**) (αhν)^2^ *vs* photon energy plots of the corresponding sample used to determine their optical band gap energy.

The electronic structure of pure and Al-doped ZnO nanoparticles was characterized by the band gap, which was essentially the energy interval between valence band and conduction band, each of which has a high density of states. We calculated the electronic band gap of undoped and Al-doped ZnO nanoparticles because of their critical role in ROS mediated cytotoxicity. Figure 3A shows the absorbance spectra of undoped and Al-doped ZnO nanoparticles. A strong absorption peak appears at 314 nm for Al-doped ZnO nanoparticles, which is significantly blue shifted corresponding to pure ZnO nanoparticles peak (351 nm). The blue shift is attributed to the Burstein-Moss effect^24^. This leads to motion of Fermi level towards conduction band due to an increase in electron concentrations from Al ions (Fig. 3B). In general, Fermi level is situated at the center of the band gap. Shifting of Fermi level depends on the type of semiconductor. Al ions (n-type semicondutor) doping increases the conductivity of the intrinsic semiconductor by adding electron energy levels near the conduction band. The electron in these energy levels can be easily excited into the conduction band (Fig. 3B).

The band gap energy (E_g_) was estimated by assuming direct transition between conduction band and valance band. Theory of optical absorption gives the relationship between the absorption coefficients α and the photon energy hν for direct allowed transition as (equation 2)^25^,


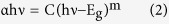


Where α is absorbance coefficient, C is constant, h is Planck’s constant, ν is photon frequency, E_g_ is optical band gap and m is 1/2 for direct band gap semiconductors.

The absorption coefficient α is defined as (equation 3);


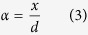


Where d is the thickness of film and x is the absorbance.

Figure 3C shows the plot of (αhν)^2^ vs hν. The linear dependence of (αhν)^2^ vs hν indicates that Al-doped ZnO nanoparticles are direct transition type semiconductor. The photon energy at the point where (αhν)^2^ is zero is E_g_. Then band gap (E_g_) is determined by the extrapolation method. The band gap for pure ZnO nanoparticles was 3.51 eV, whereas band gap of Al- doped ZnO nanoparticles was increased to 3.87 eV. The donor Al atoms provide additional carriers that causes the shifting of Fermi level towards conduction band. Therefore, band gap becomes larger.

### DLS characterization of nanoparticles

DLS characterization of pure and Al-doped ZnO nanoparticles is given in Table 1. Average hydrodynamic size of pure and Al-doped ZnO nanoparticles in cell culture medium was around 155 and 160 nm, respectively. Further, the zeta potential of pure and Al-doped ZnO nanoparticles in the same medium was −19 and −20 mV, respectively.

### Al-doping decreases dissolution of ZnO nanoparticles in culture medium

ZnO nanoparticles have a natural tendency to release Zn^2+^ ions in aqueous suspension. We have analyzed the level of Zn^2+^ ions dissolution from pure and Al-doped ZnO nanoparticles in cell culture medium. Results showed that after 24 h, Al-doped ZnO nanoparticles appear to be less soluble than pure ZnO nanoparticles (Table 2). We found that 9% of dissolution of ZnO nanoparticles as compared to only 4% dissolution of Al-doped ZnO nanoparticles. Our results were in agreement with other studies showing that metal ions doping reduce the ionization of ZnO nanoparticles^26^,^27^.

### Al-doping increased cytotoxicity of ZnO nanoparticles

**Figure 4:**
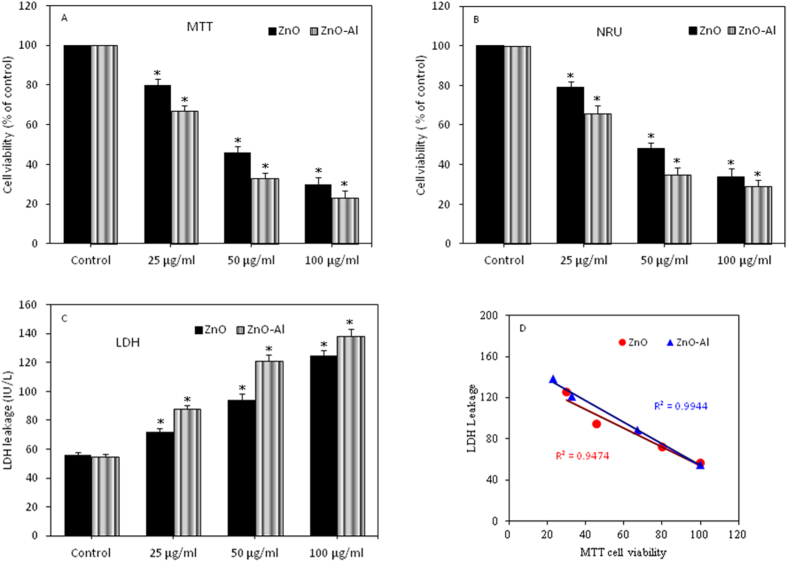
Cytotoxicity of pure and Al-doped ZnO nanoparticles in MCF-7 cells. (**A**) MTT assay (**B**) NRU assay and (**C**) LDH assay. Data represented are mean ± SD of three identical experiments made in triplicate. *Statistically significant difference as compared to controls (p < 0.05). (**D**) Significant negative correlation between MTT and LDH.

MCF-7 cells were exposed to pure and Al-doped ZnO nanoparticles at the concentrations of 25, 50 and 100 μg/ml for 24 h and cytotoxicity was determined by MTT, NRU and LDH assays. All three assays have shown that cytotoxic response of Al-doped ZnO nanoparticles was higher as compared to pure ZnO nanoparticles. MTT cell viability due to pure ZnO nanoparticles exposure was decreased to 80%, 46% and 31%, while cell viability reduction due to Al-doped ZnO nanoparticles was 67%, 33% and 22% at the concentrations of 25, 50 100 mg/ml, respectively (Fig. 4A). The IC50 estimated by MTT assay was 44 μg/ml for pure ZnO nanoparticles and 31 μg/ml for Al-doped ZnO nanoparticles. Similar trends of cell viability were observed in NRU results (Fig. 4B).

We have also found that both types of nanoparticles induced dose-dependent LDH leakage, a marker of cell membrane damage (Fig. 4C). Like cell viability, LDH leakage due to Al-doped ZnO nanoparticles exposure was higher than those of pure one. Moreover, a reverse linear correlation was observed between LDH and MTT (Fig. 4D).

### Al-doping enhanced oxidative stress response of ZnO nanoparticles

**Figure 5:**
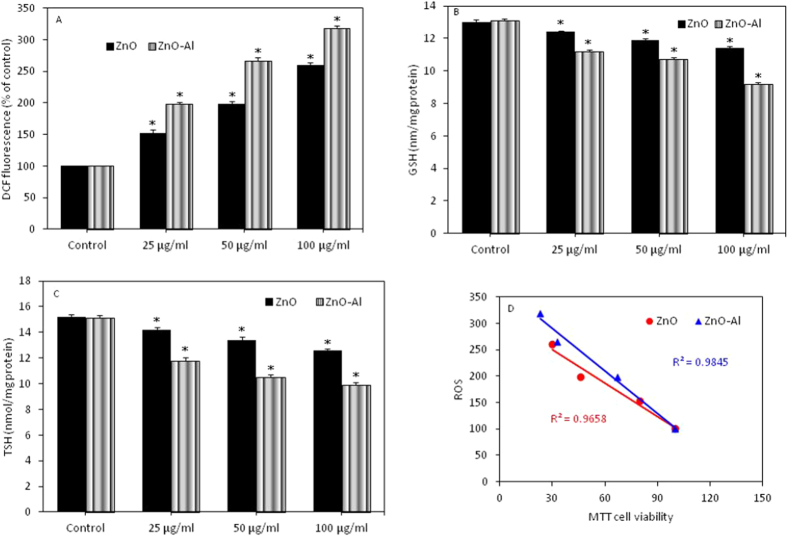
Oxidative stress response of pure and Al-doped ZnO nanoparticles in MCF-7 cells. (**A**) ROS, (**B**) GSH and (**C**) TSH. Data represented are mean ± SD of three identical experiments made in triplicate. *Statistically significant difference as compared to controls (p < 0.05). (**D**) Significant negative correlation between MTT and ROS.

Both pure and Al-doped ZnO nanoparticles were found to induce oxidative stress indicated by induction of ROS generation and depletion of glutathione (GSH) and total antioxidant (TSH) (Fig. 5A–C). Moreover, oxidative stress response of Al-doped ZnO nanoparticles was more severe than those of pure one. We also observed an inverse correlation between ROS and cell viability (Fig. 5D).

### Cytotoxicity of pure and Al-doped ZnO nanoparticles was mediated through oxidative stress

**Figure 6:**
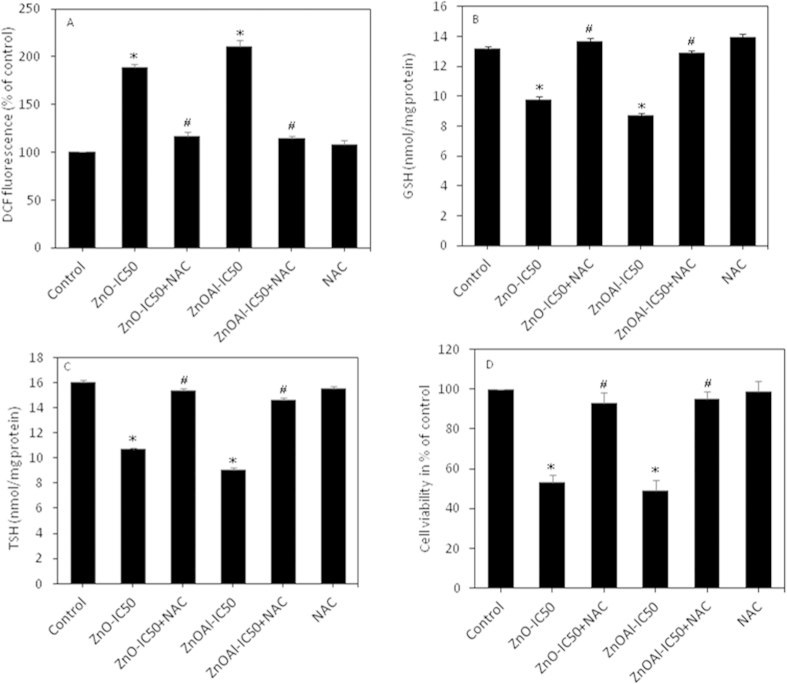
NAC prevented oxidative stress and preserved cell viability reduction caused by pure and Al-doped ZnO nanoparticles. (**A**) ROS, (**B**) GSH, (**C**) TSH and (**D**) cell viability. Data represented are mean ± SD of three identical experiments made in triplicate. *Statistically significant difference as compared to controls (p < 0.05). ^#^Significant inhibitory effect of NAC on oxidative stress and cytotoxicity (p < 0.05).

ROS generation and oxidative stress has been suggested as an explanation behind the cytotoxicity of nanoparticles^28^,^29^. In order to explore whether oxidative stress could play a key role in cytotoxicity, MCF-7 cells were exposed to both pure and Al-doped ZnO nanoparticles in the presence or absence of N-acetyl-cysteine (NAC). Results showed that NAC significantly prevented generation of ROS and depletion of antioxidants (GSH & TSH) caused by pure and Al-doped ZnO nanoparticles exposure (Fig. 6A–C). We further noticed that co-exposure of NAC, abolished almost fully the cell viability reduction caused by pure and Al-doped ZnO nanoparticles exposure (Fig. 6D). These results suggested that cytotoxicity caused by both pure and Al-doped ZnO nanoparticles was mediated through oxidative stress.

### Al-doping decreased mitochondrial membrane potential of ZnO nanoparticles

**Figure 7:**
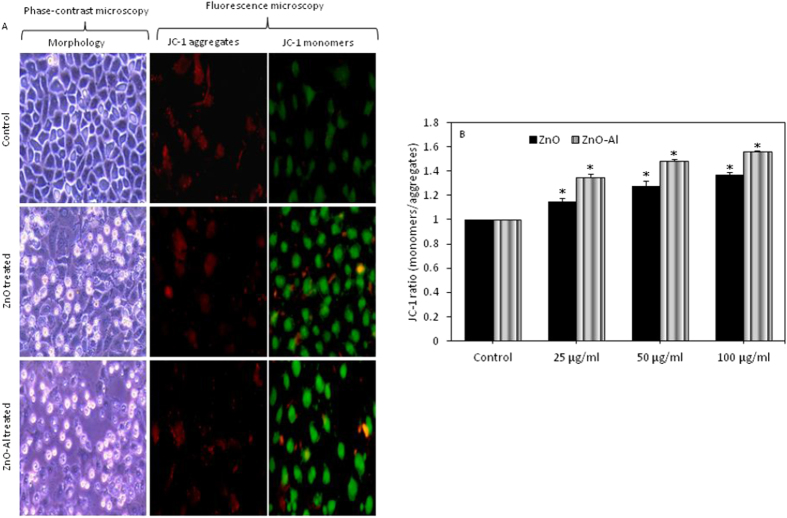
MMP loss due to pure and Al-doped ZnO nanoparticles exposure in MCF-7 cells. (**A**) Representative fluorescent image showing that pure and Al-doped ZnO nanoparticles increased the JC-1 monomers (green fluorescence) and decreased the JC-1 aggregates (red fluorescence) in MCF-7 cells as compared to control. Fluorescent images were captured with a fluorescence microscope (OLYMPUS CKX 41). Morphology of cells also supported the data of apoptosis (MMP loss) showing that treated cells detached from surface and become rounded. (**B**) JC-1 ratio (monomers/aggregates) in treated and control cells. Data represented are mean ± SD of three identical experiments made in three replicate. *Statistically significant difference as compared to the controls (p < 0.05).

Changes in MMP due to nanoparticles exposure are associated with apoptosis^30^. The MMP was evaluated after pure and Al-doped ZnO nanoparticles exposure by JC-1 probe using fluorescence microscopy and microplate reader. The green/red fluorescence intensity ratio was used to express the changes of MMP and the increased ratio indicates the MMP loss. Fluorescence microscopy showing the higher green fluorescence and lower red fluorescence in nanoparticles treated cells as compared to controls (Fig. 7A). Detachment from the surface and rounded morphology of cells due to pure and Al-doped ZnO nanoparticles exposure also supported the MMP results. Quantitative data showed the elevated level of green/red ratio (indicator of MMP loss) with the increasing of nanoparticles doses (25–100 μg/ml) (Fig. 7B). Similar to cytotoxicity and oxidative stress data, MMP loss due to Al-doped ZnO nanoparticles was higher than those of the pure ZnO nanoparticles.

### Al-doping enhanced the effect of ZnO nanoparticles apoptotic genes at transcriptional level

**Figure 8:**
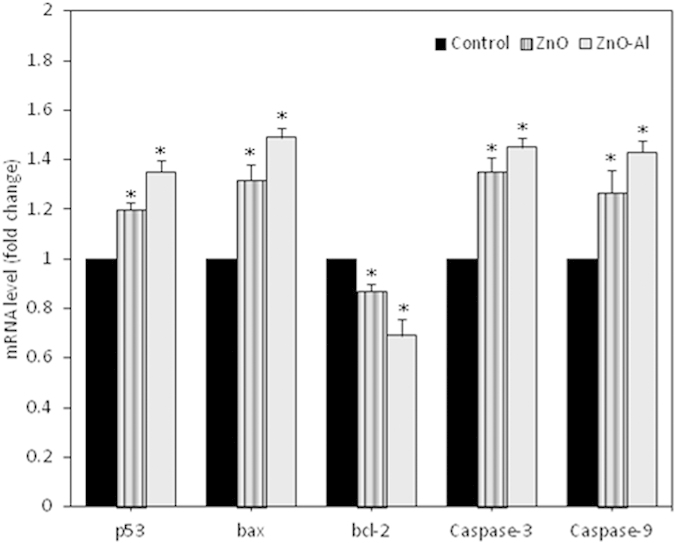
Quantitative real-time PCR analysis of mRNA levels of apoptotic genes in MCF-7 cells due to pure and Al-doped ZnO nanoparticles exposure. Data represented are mean ± SD of three identical experiments made in three replicate. *Statistically significant difference as compared to the controls (p < 0.05).

We have utilized quantitative real-time PCR to analyze the mRNA levels of apoptotic genes (p53, bax, bcl-2, caspase-3 and caspase-9) in MCF-7 cells exposed to pure and Al-doped ZnO nanoparticles at a concentration of 50 μg/ml for 24 h. Results showed that both nanoparticles significantly altered the expression levels of such genes (Fig. 8). Expression level of cell cycle checkpoint gene p53 and pro-apoptotic gene bax was up-regulated while the expression of anti-apoptotic gene bcl-2 was down-regulated in treated cells as compared to controls. We also observed the higher expression of caspase-3 and caspase-9 genes in nanoparticles exposed cells. Moreover, effect of Al-doped ZnO NPs on the expression these genes was higher than those of pure one.

### Al-doping enhanced the effects of ZnO nanoparticles on apoptotic gene at translational level

**Figure 9:**
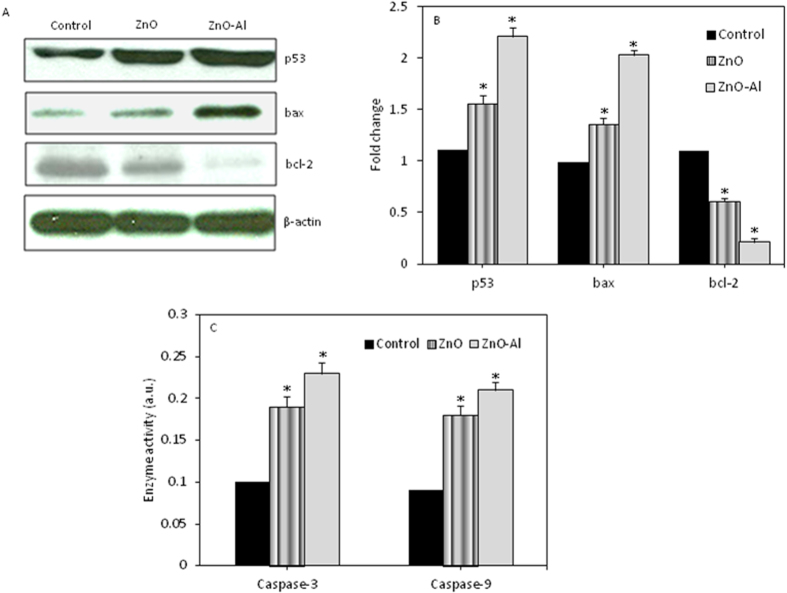
Western blot analysis of protein levels of apoptotic genes in MCF-7 cells due to pure and Al-doped ZnO nanoparticles exposure. (**A**) Immunoblot images of p53, bax and bcl-2 proteins. Blots are cropped and no other bands were observed. All the gels have run under same experimental conditions. (**B**) Protein levels were also analyzed by densitometric analysis using AlphaEase TM FC StandAlone V.4.0.0 software. Bar diagrams are from mean ± SD of three immunoblots. Results are expressed as a fold change over the control group. (**C**) Pure and Al-doped ZnO nanoparticles induced the activity of caspase-3 and caspase-9 enzymes. Enzyme data represented are mean ± SD of three identical experiments made in three replicate. *Statistically significant difference as compared to the controls (p < 0.05).

Western blotting was used to examine the expression of apoptotic genes at protein levels in MCF-7 cells exposed to pure and Al-doped ZnO nanoparticles at a concentration of 50 μg/ml for 24 h. Results showed that protein level of p53 and bax genes was up-regulated while the expression of bcl-2 was down-regulated in cells treated with pure and Al-doped ZnO nanoparticles (Fig. 9A). Densitometric analysis indicated the effect of Al-doped ZnO nanoparticles was higher as compared to pure ZnO nanoparticles supporting the transcriptional data (Fig. 9B).

### Al-doping increased caspase-3 and caspase-9 enzymes activity of ZnO nanoparticles

We further determined the activity of apoptotic enzymes (caspase-3 & caspas-9) in MCF-7 cells exposed to pure and Al-doped ZnO nanoparticles at a concentration of 50 μg/ml for 24 h. Results demonstrated that activity of both enzymes was higher in cells treated with pure and Al-doped ZnO nanoparticles (Fig. 9C). Similarly, effect of Al-doped ZnO nanoparticles on both types of apoptotic enzymes was greater than those of the pure one.

### Al-doping did not change the benign nature of ZnO nanoparticles toward normal cells

**Figure 10:**
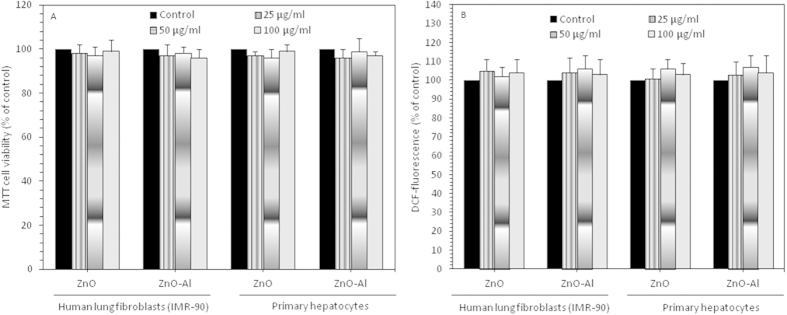
Effect of pure and Al-doped ZnO nanoparticles on viability of human lung fibroblasts (IMR-90) and primary rat hepatocytes. Data represented are mean ± SD of three identical experiments made in triplicate. *Statistically significant difference as compared to controls (p < 0.05).

To examine whether Al-doping change the benign nature of ZnO nanoparticles, we determined the effect of pure and Al-doped ZnO nanoparticles on normal human lung fibroblasts (IMR-90) and primary rat hepatocytes. Results have shown that Al doping did not change the inherent selective cytotoxicity nature of ZnO nanoparticles by posing no effects (cytotoxicity and ROS generation) on IMR-90 and rat hepatocytes (Fig. 10A,B). These results suggested that Al-doping improves the killing ability of ZnO nanoparticles toward human breast cancer cells without affecting the normal cells.

## Discussion

Physicochemical characterization of nanoscale materials is imperative before their biomedical studies^29^,^31^. Size, shape, crystallinity, purity and aqueous behavior of pure and Al-doped ZnO nanoparticles were characterized. Both types of nanoparticles have almost similar crystallite size and surface morphology. We noticed that secondary particle size (hydrodynamic size) of both types of nanoparticles were 3–4 times higher than primary particle size (size of dry powder) (Table 1). The higher size of nanoparticles in aqueous state than primary size could be due to tendency of particles to agglomerate in aqueous suspension^32^. That indicates the possible interaction of nanoparticles with the protein of culture media, which has been widely reported with different type of nanoparticles that leads to the formation of protein corona^4^,^31^. Therefore, not only the primary size but also the secondary size of nanoparticles could be used as a characteristic parameter in biological investigations.

The key finding of characterization study was that Al-doping increased the band gap energy of ZnO nanoparticles (from 3.51 eV for pure to 3.87 eV for Al-doped ZnO). The addition of donor impurities (e.g. Al) contributes electron energy levels high in semiconductor band gap so that electrons can be easily excited into conduction band, which causes Fermi level to be shifted towards conductions band (blue shift). Promotion of electrons (e^−^) across the band gap to conduction band creates a hole (h^+^) in valence band. The holes are powerful oxidants and they can react with water or surface-bound chemisorbed hydroxyl groups to produce hydroxyl radicals. The conduction band electrons are good reducing agents, and can move to the particle surface and be trapped in metastable surface states, or react with electron acceptors or oxidants such as adsorbed O_2_ (Fig. 3). This could be one of the possible explanations behind our results showing that Al-doping enhanced the cytotoxic and oxidative response of ZnO nanoparticles in MCF-7 cells. The wide electronic band gap structures of metal oxides nanoparticles with the redox potentials of different ROS generation reactions have also been proposed^33^,^34^,^35^. Recent studies suggested that very limited research has been done on the role of the electronic band gap behavior of metal-oxide nanoparticles in ROS mediated toxicity^36^.

Cytotoxicity parameters such as MTT, NRU and LDH assays represent the damage in mitochondrial, lysosomal and cell membranes, respectively that eventually triggers cell death^37^. These assays serve as sensitive and integrated measures of cell integrity and of the inhibition of cell proliferation. Our cytotoxicity results were consistent with the observed higher MMP loss in cells treated with Al-doped ZnO nanoparticles than those of pure ZnO nanoparticles. Damage to lysosomal membranes is known to release lysosome protease into intracellular spaces, which affects the neighboring cells and triggers cell death due to apoptosis. LDH leakage from cells is further evidence for the penetration of nanoparticles into cells and cell membrane damage^16^,^38^.

Apoptosis is a critical process in the development and progression of cancer. The ability of cancer cells to avoid apoptosis and continue to propagate is one of the basic characteristics of cancer and is also a major target for cancer therapy^39^. We have provided the evidence that Al-doped ZnO nanoparticles induce apoptosis in MCF-7 cells. Expression of both mRNA and protein levels of cell cycle checkpoint gene p53 and pro-apoptotic gene bax were up-regulated, while the expression of anti-apoptotic gene bcl-2 was down-regulated in cells treated with Al-ZnO nanoparticles as compared to controls. It has been suggested that bax is up-regulated by p53 gene^40^. Since an increase in bax expression was observed, the role of p53 in the up-regulation of bax upon Al-doped ZnO nanoparticles exposure can be postulated. The insertion of bax into the mitochondrial membrane possibly leads to p53-mediated apoptosis^40^. Caspases are activated during apoptosis in many cells and are known to play a vital role in both initiation and execution of apoptosis. We also observed the higher activity of caspase-3 and caspase-9 enzymes in Al-doped ZnO nanoparticles treated cells than those of controls. Taken together, up-regulation of p53 leads to activation of pro-apoptotic members of bcl-2 family, such as bax induces permeabilization of the outer mitochondrial membrane, which releases soluble proteins from the intermembrane space into the cytosol, where they promote caspase activation^41^. The best studied of these proteins is cytochrome c, which binds to apoptosis protease activating factor-1 (Apaf-1) and leads to the assembly of an apoptosome complex. This apoptosome can bind procaspase-9 and cause its autoactivation through a conformational change. Once initiated caspase-9 goes on to activate caspase-3 (effector caspase), which cleaves substrates at aspartate residues and activation of this proteolytic activity appears to be an event in apoptosis^42^. Our apoptotic data also supported the cytotoxicity and oxidative stress data that Al-doped ZnO nanoparticles induce higher apoptotic response than those of pure one.

Some studies argued that ZnO nanoparticles induce toxicity due to the dissolution of the particles into Zn^2+^ ions and metal ions doping can reduce the ZnO cytotoxicity by reducing ZnO dissolution^26^,^27^. However, some investigators demonstrated that ZnO nanoparticles liberate Zn^2+^ ions in aqueous state, but the levels of Zn^2+^ions released were insufficient to promote cytotoxicity in human cells unless the particulate matter is in contact with the cells^43^. In the present study, we also observed that Al-doping significantly reduce the ionic dissolution ZnO nanoparticles (Table 2). We infer that strong bonding of Al in the host lattice of ZnO is responsible for the significant reduction of ZnO nanoparticle dissolution. We also found that released Zn^2+^ions from the surface of ZnO nanoparticles did not cause much toxicity to MCF-7 cells (data not shown). Hence, we believe that the primary mechanism of cytotoxicity induced by ZnO nanoparticles involved catalytic destruction of biomolecules due to induction of ROS generation.

One of the important findings of this study was that Al-doping did not change the benign nature of ZnO nanoparticles towards normal cells. We found that pure and Al-doped ZnO nanoparticles did not induce cytotoxicity and intracellular ROS generation in normal human lung fibroblast (IMR-90) and primary rat hepatocytes (Fig. 10). These results suggest that Al-doping improves the selectively killing ability of ZnO nanoparticles towards cancer cells (MCF-7) without posing much impact on normal cells. Why Al-doped ZnO nanoparticles kill cancers via ROS generation and spare the normal cells? Understanding the biochemical differences between cancer and normal cells can helps a lot in this approach. Cancer cells as compared to normal cells are under greater intrinsic oxidative stress due to alterations in metabolism and higher production of ROS^44^. Higher level of ROS in cancer cell may have useful consequences such as induction of cell propagation. ROS are chemically very active and may impose serious oxidative damage to cell biomolecules and provide an opportunity to destroy cancer cells according to the susceptibility of ROS level^45^. Consequently, higher level of ROS might act as a double-edged sword. A moderate increase of ROS could trigger cell division and differentiation, as well as other features of cancer. But, when ROS concentrations increase up to the lethal level it may break through the antioxidant capacity of the cell and trigger apoptosis. In normal physiological situation, normal cells maintain redox homeostasis with a low level of ROS by controlling the equilibrium between the production of ROS and their removal by antioxidants^46^. Hence, manipulating ROS generation by Al-doped ZnO nanoparticles is a potential way to kill MCF-7 cancer cells selectively without imposing much toxicity to normal cells.

## Conclusion

We found that Al-doping improves the inherent selective cytotoxicity of ZnO nanoparticles toward human breast cancer cells while posing no toxicity to normal cells. The improved cytotoxic response ZnO nanoparticles due Al-doping was related to widening of band gap energy that increased their ability to induce ROS generation. Furthermore, Al-doped ZnO nanoparticles were found to induce apoptosis in MCF-7 cells. Tumor suppressor gene p53 was up-regulated due to Al-doped ZnO nanoparticles exposure. Decrease in MMP with a concomitant increase in bax/bcl2 ratio suggested that Al-doped ZnO nanoparticles induced apoptosis through mitochondrial pathway. Overall, our data suggested a novel approach through which the inherent selective cytotoxicity of ZnO nanoparticles against human breast cancer cells can be improved. Further research on anticancer activity of Al-doped ZnO nanoparticles in different types of cancer cells is warranted.

## Materials

Dulbecco’s modified eagle’s medium (DMEM), hank’s balanced salt solution (HBSS), fetal bovine serum (FBS), penicillin-streptomycin and trypsin were purchased from Invitrogen Co. (Carlsbad, CA, USA). Zinc nitrate hexahydrate [Zn(NO_3_)_2_.6H_2_O], 3-(4,5–2-yl)-2,5-diphenyltetrazoliumbromide (MTT), 3-amino-7-dimethylamino-2-methyl-phenazine hydrochloride (neutral red), 5,5′ 6,6′-tetrachloro-1,1′ 3,3′- tetraethylbenzimi dazo-lylcarbocyanide iodine dye (JC-1), 5,5-dithio-bis-(2-nitrobenzoic acid) (DTNB), 2,7-dichlorofluorescin diacetate (DCFH-DA), glutathione (GSH), N-acetyl-cystein (NAC), anti-p53 antibody, anti-bax antibody, anti-bcl-2 antibody and anti-β-actin antibody were obtained from Sigma-Aldrich (St. Louis, MO, USA). Secondary antibodies and RIPA buffer were purchased from Santa Cruz Biotechnology, Inc. (Santa Cruz, CA, USA). Caspase-3 and caspase-9 enzymes assay kits were purchased from Bio-Vision Inc. (Milpitas, California, USA). All other chemicals used were of the highest purity available from the commercial sources.

## Methods

### Synthesis of nanoparticles

Zinc nitrate hexahydrate [Zn(NO_3_)_2_.6H_2_O] was used as a precursor for ZnO nanoparticles synthesis using sol-gel method as described in our recent publication^11^. Sol-gel method can be employed for the preparation of ZnO nanoparticles from zinc nitrate in addition to zinc acetate^47^. ZnO nanoparticles prepared from zinc nitrate exhibit fine grain of nanoparticles. It is also found that ZnO nanoparticles prepared from nitrate show a rapid crystallization compared to ZnO nanoparticles produced from zinc acetate. To prepare ZnO nanoparticles, 1 M zinc nitrate was mixed with the appropriate amount of stabilizer ethylene glycol (CH_2_OH)_2_ in de-ionized water. For aluminum (Al) doped ZnO nanoparticles preparation, aluminum nitrate nanohydrate (Al(NO_3_)_3_.9H_2_O) was added into solution to serve as Al source. The amount of Al(NO_3_)_3_.9H_2_O was controlled precisely and kept at the selected concentration of Al. The Al atomic contents of 3 atwt % were chosen, and then stirred in clear solution at 70 °C for 90 seconds. Meanwhile, citric acid (C_6_H_8_O_7_) was dropped into this solution slowly until the solution had a uniform distribution and became transparent (pH ~ 2). The solution was then aged for 48 h to assure that the chemical process was completed. After ageing, solution was placed in an oven at 120 °C until the solvent evaporated; and then the dried samples were grinded into nanopowder using pestle and mortar.

### Characterization of nanoparticles

Crystalline nature of pure and Al-doped ZnO nanoparticles was measured by XRD technique. XRD pattern of nanopowder was acquired at room temperature with the help of a PANalytical X’Pert X-ray diffractometer equipped with an Ni filter using Cu Kα (λ = 1.54056 Å) radiations as an X-ray source. Morphology of both nanoparticles was determined by field emission transmission electron microscopy (FETEM) (JEM-2100F, JEOL Inc,) at an accelerating voltage of 200 kV. Energy dispersive X-ray spectroscopy (EDS) was performed for compositional analysis of the samples. The UV-visible analysis was carried out in the wavelength range of 200–1200 nm using a double beam UV-vis-NIR spectrophotometer (Thermo scientific evolution 60 S) with a resolution of 0.5 nm.

Hydrodynamic size and zeta potential of pure and Al-doped ZnO nanoparticles in complete cell culture medium (DMEM with 10% FBS) were determined by dynamic light scattering (DLS) (Nano-ZetaSizer-HT, Malvern Instruments, Malvern, UK) as described by Murdock *et al.*^48^. Briefly, pure and Al-doped ZnO nanoparticles were suspended in culture medium at a concentration of 100 μg/ml for 24 h. Then, suspensions were sonicated using a sonicator bath at room temperature for 15 minutes at 40 W and DLS experiments were performed.

Dissolution experiments of pure and Al-doped ZnO nanoparticles were performed in a glass beaker at room temperature. In beaker we have prepared different concentrations (25, 50 and 100 μg/ml) of pure and Al-doped ZnO nanoparticles in culture medium. Suspensions were incubated for 24 h. Then, aliquots from the supernatant were collected from the beaker, and solid components were removed by centrifugation. From these samples concentration of Zn^2+^ ions was measured by inductively coupled plasma mass spectrometry (ICP-MS). The mean ± SD of triplicate measures was reported for all dissolution measurements.

### Cell Culture and exposure to nanoparticles

Human breast cancer cells (MCF-7) and two types of normal cells (human lung fibroblasts IMR-90 and primary rat hepatocytes) were used to determine cytotoxicity of pure and Al-doped ZnO nanoparticles. The MCF-7 and IMR-90 cells were obtained from American Type Culture Collection (ATCC) (Manassas, VA, USA). Primary rat hepatocytes were isolated by collagenase perfusion technique as described by Moldeus^49^.

Cells were cultured in DMEM or RPMI1640 medium supplemented with 10% FBS and 100 U/ml penicillin-streptomycin at 5% CO_2_ and 37 °C. Cells were routinely cultured in 25 cm^2^ culture flask and passage for every 2–4 days before reaching the confluence level. At 85% confluence, cells were harvested using 0.25% trypsin and then sub-cultured into 25 cm^2^ culture flask, 6-well plate or 96-well plate according to the selection of experiments. Cells were allowed to attach the surface for 24 h prior to nanoparticles exposure. Pure and Al-doped ZnO nanoparticles were suspended in culture medium and diluted to appropriate concentrations (25, 50 and 100 μg/ml). Dilutions of nanoparticles were then sonicated using a sonicator bath at room temperature for 15 min at 40 W to avoid nanoparticles agglomeration prior to exposure of cells. Selection of 25–100 μg/ml dosages was based on a preliminary dose-response study (data not shown). Under some conditions, MCF-7 cells were pre-exposed for 1 h with 10 mM of N-acetyl-cystein (NAC) before 24 h co-exposure with or without nanoparticles. Cells not exposed to nanoparticles served as control in each experiment.

### MTT assay

MTT assay was carried out following the method as described by Mossman^50^ with some modifications^51^. The MTT assay assesses mitochondrial function by measuring ability of viable cells to reduce MTT into blue formazon product. In brief, 1 × 10^4^ cells/well were seeded in 96-well plates and exposed to different concentrations of pure and Al-doped ZnO nanoparticles for 24 h. At the end of the exposure time, culture medium was removed from each well to avoid the interference of nanoparticles and replaced with new culture medium containing MTT solution in an amount equal to 10% of culture volume, and incubated for 3 h at 37 °C until a purple-colored formazan product developed. The resulting formazan product was dissolved in acidified isopropanol. Following this, each 96-well plate was centrifuged at 2,300 g for 5 min to settle down the remaining nanoparticles present in the solution. Then, 100 μl supernatant was transferred to the fresh wells of new 96-well plate and absorbance was measured at 570 nm using a microplate reader (Synergy-HT, BioTek, USA).

### Neutral red uptake assay

Neutral red uptake (NRU) assay was performed following the procedure as described by^52^ with some specific modifications^51^. In brief, 1 × 10^4^ cells/well were seeded in 96-well plates and exposed to different concentrations of pure and Al-doped ZnO nanoparticles for 24 h. At the end of the exposure time, the test solution was aspirated and cells were washed with phosphate buffer saline twice before being incubated for 3 h in medium supplemented with neutral red (50 μg/ml). The medium was washed off rapidly with a solution containing 0.5% formaldehyde and 1% calcium chloride. The cells were then incubated for a further 20 min at 37 °C in a mixture of acetic acid (1%) and ethanol (50%) to extract the dye. The 96-well plates were then centrifuged at 2,300 g for 5 minutes to settle the remaining NPs present in the solution. Following this, 100 μl of the supernatant was transferred to new 96-well plates and the absorbance was measured at 540 nm using a microplate reader (Synergy-HT, BioTek, USA).

### Lactate dehydrogenase assay

Lactate dehydrogenase (LDH) is an enzyme widely present in cytosol that converts lactate into pyruvate. When plasma membrane integrity is ruptured, LDH leaks into culture media and its extracellular level is increased. LDH assay was carried out with the method described earlier^53^ with some modifications^51^. In brief, 1 × 10^4^ cells/well were seeded in 96-well plate and exposed to different concentrations of pure and Al-doped ZnO nanoparticles for 24 h. At the end of the exposure time, 96-well plate was centrifuged at 2,300 g for 10 minutes to get the cell culture media. Then, 100 μl of culture media transferred to new fresh tube containing 100 μl of sodium pyruvate (prepared in 2.5 mg/ml phosphate buffer) and 100 μl of reduced nicotinamide adenine dinucleotide (NADH) (prepared in 2.5 mg/ml phosphate buffer) in a total volume of 3.0 ml (0.1 M potassium phosphate buffer, pH 7.4). The rate of NADH oxidation was determined by following the decrease in absorbance at 340 nm for 3 minutes at 30 seconds interval using a spectrophotometer (Thermo-Spectronic, Genesys 10; Spectronic Unicam, Rochester, NY, USA). The amount of LDH released is represented as LDH activity (IU/l) in culture media.

### Measurement of reactive oxygen species

Intracellular ROS level was measured using 2,7-dichlorofluorescin diacetate (DCFH-DA)^54^ with some specific modifications^51^. The DCFH-DA passively enters the cell, where it reacts with ROS to form the highly fluorescent compound dichlorofluorescein (DCF). Briefly, cells (1 × 10^4^ cells/well) were seeded in 96-well black-bottomed culture plates and allowed to adhere for 24 h in a CO2 incubator at 37 °C. Next, the cells were exposed to different concentrations of pure and Al-doped ZnO nanoparticles for 6 h and 24 h. At the end of the exposure time, cells were washed twice with HBSS before being incubated in 1 ml of working solution (100 μM) of DCFH-DA at 37 °C for 30 minutes. Following this, the cells were lysed in alkaline solution and centrifuged at 2300 g for 10 minutes. A 200 μl amount of the supernatant was transferred to a new 96-well plate, and fluorescence was measured at 485 nm excitation and 520 nm emission using the microplate reader (Synergy-HT, BioTek, USA). The values were expressed as a percent of fluorescence intensity relative to the control wells.

### Assay of glutathione and total thiol

GSH level was quantified by fluorometric assay as described by Hissin and Hilf^55^. Briefly, cells were cultured in 6-well plates and exposed to different concentrations of undoped and Al-doped ZnO nanoparticles for 24 h. At the end of the exposure time, the cells were washed twice in cold phosphate buffer saline and lysed in distilled water containing deoxycholic acid plus sucrose by four cycles of freeze-thaw and centrifuged at 10,000 g for 10 minutes at 4 °C. The supernatant was transferred to another tube and total thiol (TSH) (as described later for GSH) and protein content was measured. For the determination of intracellular GSH, protein in this supernatant was precipitated at 0.25% trichloroacetic acid and again centrifuged at 10,000 g for 5 minutes at 4 °C. Then, 20 μl amount from the protein precipitated sample was mixed with 160 μl of 0.1 M phosphate-5 mM EDTA buffer (pH 8.3) and 20 μl of O-phthalaldehyde (OPT, 1 mg/ml in methanol) in a black 96-well plate. After 2 h of incubation at room temperature in dark, fluorescence was measured at emission wavelength of 460 nm and excitation wavelength of 350 nm. GSH standard was prepared in 0.25% trichloroacetic acid. Results are expressed as TSH nmol/mg protein and GSH nmol/mg of cellular protein.

### Cell morphology

Morphology of MCF-7 cells following exposure to pure and Al-doped ZnO nanoparticles was examined by phase-contrast microscopy (Leica DMIL, Germany).

### Mitochondrial membrane potential assay

Changes in mitochondrial membrane potential (MMP) due to pure and Al-doped ZnO nanoparticles exposure were estimated by JC-1 dye. This probe (JC-1) can selectively enter into mitochondria and reversibly change color from red (aggregates) to green (monomer) as the membrane potential decreased^56^. MMP was measured by two methods; quantitative analysis by microplate reader and qualitative analysis by microscopic fluorescence imaging. For microplate reader assay, 1 × 10^4^ cells/well were seeded in 96-well plates and exposed to different concentrations of undoped and Al-doped ZnO nanoparticles for 24 h. At the end of the exposure time, cells were washed with PBS and incubated with 3 μM JC-1 solution (prepared in PBS) for 30 minutes. Following this, cells were washed twice with phosphate buffer saline and measured by the microplate reader (Synergy-HT, BioTek, USA). The green fluorescence (monomer) intensity was determined at an excitation wavelength of 485 nm and an emission wavelength of 535 nm, whereas the red fluorescence (aggregates) intensity determined at an excitation wavelength of 560 nm and an emission wavelength of 595 nm. The ratio of fluorescence intensity of monomer (green) to fluorescence intensity of aggregates (red) was used as indicator of MMP. A parallel set of appropriate cells in 6-well plates were analyzed for fluorescence imaging using a fluorescence microscope (OLYMPUS CKX 41) by capturing the images at 20X magnification.

### Quantitative real-time PCR analysis

Cells were cultured in 6-well plates and exposed to 50 μg/ml of pure and Al-doped ZnO nanoparticles for 24 h. At the end of the exposure time, total RNA was extracted by RNeasy mini Kit (Qiagen,Valencia, CA, USA) according to the manufacturer’s instructions. Concentration of the extracted RNA were determined using Nanodrop 8000 spectrophotometer (Thermo-Scientific, Wilmington, DE, USA) and the integrity of RNA were visualized on 1% agarose gel using gel documentation system (Universal Hood II, BioRad, Hercules, CA). The first strand cDNA was synthesized from 1 μg of total RNA by Reverse Transcriptase using M-MLV (Promega, Madison, WI) and oligo (dT) primers (Promega) according to the manufacturer’s protocol. Quantitative real-time PCR (RT-PCRq) was performed by QuantiTect SYBR Green PCR kit (Qiagen) using ABI PRISM 7900HT Sequence Detection System (Applied Biosystems, Foster City, CA). Two microliter of template cDNA was added to the final volume of 20 μl of reaction mixture. Real-time PCR cycle parameters included 10 min at 95 °C followed by 40 cycles involving denaturation at 95 °C for 15 s, annealing at 60 °C for 20 s and elongation at 72 °C for 20 s. The sequences of the specific sets of primer for p53, bax, bcl-2, caspase-3, caspase-9 and β-actin used in this study are given in our earlier published paper [32]. Expressions of selected genes were normalized to β-actin gene, which was used as an internal housekeeping control. All the real-time PCR experiments were performed in triplicate and data expressed as the mean of at least three independent experiments.

### Western blotting

Cells were cultured in 6-well plates and exposed to 50 μg/ml of pure and Al-doped ZnO nanoparticles for 24 h. The harvested cell pellets were lysed in RIPA lysis buffer [1X TBS (0.5 M Tris-HCl and 1.5 M NaCl) pH 7.4, 1% NP-40, 0.5% sodium deoxycholate, 0.1% SDS, 0.004% sodium azide] in the presence of a protease inhibitor. The cell lysates were then analyzed for protein content using SDS-Page immunoblotting. The membrane was then probed with p53, bax, bcl-2 and β-actin antibodies to determine the expression of proteins. The β-actin was used as a loading control. Protein levels were also analyzed by desitometric analysis using AlphaEase TM FC StandAlone V.4.0.0 software. Results are expressed as a fold change over the control group.

### Assays of caspase-3 and caspase-9 enzymes

Activity of caspase-3 and caspase-9 enzymes was measured in cells treated with 50 μg/ml of pure and Al-doped ZnO nanoparticles for 24 h using commercial kit (BioVision Inc., Milpitas, CA, USA). This assay is based on the principle that activated caspases in apoptotic cells cleave the synthetic substrates to release free chromophore p-nitroanilide (pNA), which is measured at 405 nm. The pNA was generated after specific action of caspase-3 and caspase-9 on tertrapeptide substrates DEVD-pNA and LEHD-pNA, respectively. In brief, the reaction mixture-consisting of 50 μl of cell extract protein (50 μg) (from control and treated cells), 50 μl of 2X reaction buffer (containing 10 mM dithiothreitol) and 5 μl of 4 mM DEVD-pNA (for caspase-3) or LEHD-pNA (for caspase-9) substrate in a total volume of 105 μl- was incubated at 37 °C for 1 h. Then the absorbance of the product was measured using the microplate reader (Synergy-HT, BioTek) at 405 nm according to manufacturer’s instruction.

### Estimation of protein

Protein content in cell extracts was estimated by Bradford method^57^ using bovine serum albumin as the standard.

### Statistical analysis

Statistical significance was determined by one-way analysis of variance (ANOVA) followed by Dunnett’s multiple comparison test. Significance was ascribed at p < 0.05. All analyses were conducted using the Prism software package (GraphPad Software, Version 5.0, GraphPad Software Inc., San Diego, USA).

## Additional Information

**How to cite this article**: Akhtar, M. J. *et al.* Aluminum doping tunes band gap energy level as well as oxidative stress-mediated cytotoxicity of ZnO nanoparticles in MCF-7 cells. *Sci. Rep.* **5**, 13876; doi: 10.1038/srep13876 (2015).

## Figures and Tables

**Table 1:** Physicochemical characterization of pure and Al-doped ZnO nanoparticles.

**Parameters**	**Pure ZnO nanoparticles (mean value)**	**Al-doped ZnO nanoparticles (mean value)**
XRD size (nm)	41	39
TEM size (nm)	43	40
Hydrodynamic size in CDMEM (nm)	155	160
Zeta potential in CDMEM (mV)	−19	−20
Elemental impurities (by EDS spectra)	ND	ND

CDMEM; Complete cell culture medium (DMEM with 10% FBS). ND; Not detected.

**Table 2:** The extent of Zn^2+^ released in cell culture medium from pure and Al-doped ZnO nanoparticles.

**Sample**	**Amount of Zn**^**2+**^ **ions released, mean ± SD (%)**		
	**25 μg/ml**	**50 μg/ml**	**100 μg/ml**
Pure ZnO nanoparticles	2.16 ± 0.17 (8.7%)	4.22 ± 0.21 (8.4%)	8.84 ± 0.19 (8.8%)
Al-doped ZnO nanoparticles	1.11 ± 0.23 (4.4%)	2.12 ± 0.12 (4.2%)	4.34 ± 0.20 (4.3%)
